# Losing Ground - Swedish Life Expectancy in a Comparative Perspective

**DOI:** 10.1371/journal.pone.0088357

**Published:** 2014-02-06

**Authors:** Sven Drefahl, Anders Ahlbom, Karin Modig

**Affiliations:** 1 Demography Unit, Department of Sociology, Stockholm University, Stockholm, Sweden; 2 Institute of Environmental Medicine, Division of Epidemiology, Karolinska Institutet, Stockholm, Sweden; University of Washington, United States of America

## Abstract

**Background:**

In the beginning of the 1970s, Sweden was the country where both women and men enjoyed the world's longest life expectancy. While life expectancy continues to be high and increasing, Sweden has been losing ground in relation to other leading countries.

**Methods:**

We look at life expectancy over the years 1970–2008 for men and women. To assess the relative contributions of age, causes of death, and smoking we decompose differences in life expectancy between Sweden and two leading countries, Japan and France. This study is the first to use this decomposition method to observe how smoking related deaths contribute to life expectancy differences between countries.

**Results:**

Sweden has maintained very low mortality at young and working ages for both men and women compared to France and Japan. However, mortality at ages above 65 has become considerably higher in Sweden than in the other leading countries because the decrease has been faster in those countries. Different trends for circulatory diseases were the largest contributor to this development in both sexes but for women also cancer played a role. Mortality from neoplasms has been considerably low for Swedish men. Smoking attributable mortality plays a modest role for women, whereas it is substantially lower in Swedish men than in French and Japanese men.

**Conclusions:**

Sweden is losing ground in relation to other leading countries with respect to life expectancy because mortality at high ages improves more slowly than in the leading countries, especially due to trends in cardiovascular disease mortality. Trends in smoking rates may provide a partial explanation for the trends in women; however, it is not possible to isolate one single explanatory factor for why Sweden is losing ground.

## Introduction

Life expectancy in the most advanced countries has increased considerably over the last 160 years or more. At the beginning the increase was fueled by reductions in infant and child mortality, but since the 1950s most part of the mortality reductions were observed for older ages [1].

**Figure 1:**
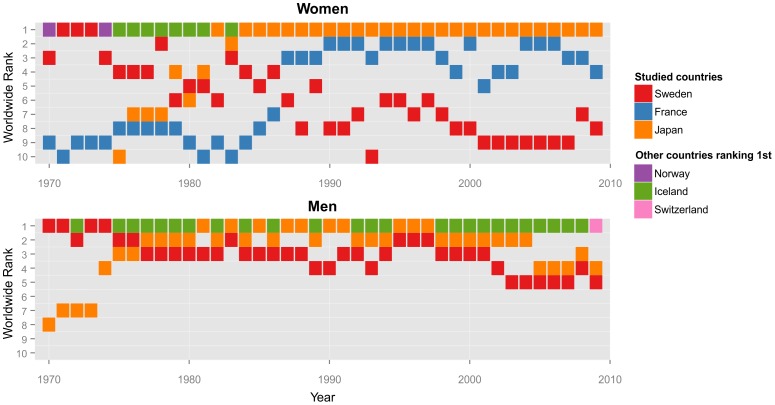
Leading Countries in Life Expectancy at Birth and Swedish World Rank (1970–2009).

Statistics on Swedish mortality can be followed for more than 250 years back in time. Over that period, life expectancy has increased from 40 to 80 years, by an average of 2 months per year. Sweden had during a long period the lowest mortality rates in the world among young individuals and was among the top three for total life expectancy. In the beginning of the 1970s, Sweden was the country where both women and men enjoyed the world's longest expectation of life (see Figure 1). In the past two decades, however, this pattern has changed.

While still among the countries with very high life expectancy [2], Sweden as well as other Nordic countries is quite rapidly losing ground in relation to other leading countries. Those countries such as Japan and France have made exceptionally fast progress in life expectancy, especially for women (Figure 1). Life expectancy at birth continues to increase in Sweden at a steady pace but mortality improvements at the very oldest ages have stagnated [3], [4], while they continue in many other countries [5].

Circulatory diseases and neoplasms are the most important broad causes of death. For both men and women mortality from circulatory diseases has decreased quite fast in the last decades. For Sweden, studies have shown reductions for acute myocardial infarction (average annual decrease in incidence by about 4% and case fatality by about 5% for both sexes combined between 1994 and 2010), coronary heart disease (mortality reduction of 53.4% in men and 52% in women aged 25–84 between 1986 and 2002), and all cardiovascular diseases (average annual decrease in incidence in the age group 65–85 by about 2% and case fatality by about 2% for both sexes combined between 1994 and 2010) [6], [7]. Mortality from cancers has decreased as well but the speed of decrease has been considerably slower [8]. In Sweden, age-standardized mortality rates for neoplasms decreased annually by 0.6% for men and 0.5% for women on average at all ages (world standard) [8]. Among women, lung cancer has been the leading cause of age standardized deaths due to cancer in Sweden in 2012 [9]. Among men, lung cancer is the leading cause of cancer death among all European countries, except Sweden, where prostate cancer has been the leading cause [9]. In fact, Sweden has the lowest lung cancer rates among men of all developed countries, while rates are about average for women [10]. The difference between men and women for this important type of cancer is likely linked to the rather unique Swedish smoking pattern. Despite similar smoking rates for men and women within Sweden [11], men have very low smoking rates relative to other countries but women do not. This sex difference was observed already in the 1970s when the smoking prevalence of Swedish men was among the lowest of all countries, while the prevalence among Swedish women was above average for countries with available data [11]. At the same time, Sweden has one of the lowest sex differences in mortality compared to other countries [12].

This paper explores the trends and differences in life expectancy by decomposing differences in life expectancy between populations. We study how mortality in different age groups, broad causes of death, and smoking attributed mortality contribute to life expectancy differences. To assess these differences we compare the Swedish mortality with the pattern observed in two of the world's leading countries, France and Japan. Japan is the world's leader in terms of life expectancy since the beginning of the 1980s. France is the leading country in Europe. All three countries have high quality-national level data for all-cause and cause specific mortality.

## Materials and Methods

In the first part of the analysis we use period life-tables by 5-year age groups separated for men and women. The data come from the Human Mortality Database, which contains original calculations of death rates and life tables for countries where death registration and census data is virtually complete [13]. We apply a decomposition technique first proposed by Arriaga [14], calculating the contribution of each age group to the overall difference in life expectancy between two countries. The individual contribution of a mortality change between ages *x* and *x+n* is calculated as the change in life expectancy that we would observe if we replace the mortality rate between ages *x* and *x +n* of country A with the mortality rate between ages *x* and *x+n* of country B, keeping the mortality rates at all other ages the same. The sum of the contributions of each age group is the overall difference in life expectancy between the two countries. The calculations are done for each single year between 1970 and 2009, separately for men and women. The decomposition formula provided by Ariaga using the notation from Preston et al. [15] is provided in Appendix S1.

In the second part of the analysis we examine how broad groups of causes of death contribute to the life expectancy differences. We expand the decomposition techniques described above with an extension for estimating the contribution of specific causes of death to the differences in life expectancy also proposed by Arriaga [16]. The calculation is based on the idea that each age group specific contribution to life expectancy differences can be subdivided into separate causes of death. To obtain the age-specific contribution in years of a cause of death to the overall difference in life expectancy one would first calculate the absolute difference in the specific cause of death mortality rate between the two countries for ages *x* to *x+n*. Dividing this number by the absolute difference of the all-cause mortality rates between the two countries in the same age group yields the proportion of the overall difference in the mortality rates between ages *x* and *x+n* that is due to the difference in the mortality rates of a cause of death in that age group. This proportion is then multiplied with the total age-group specific contribution obtained in the first part of the analysis. The formula for the extension is given in Appendix S1.

The cause specific mortality rates are obtained from the WHO Mortality Database [17]. The database includes annual data on all deaths registered in national vital registration systems, separated by underlying cause of death and age group. For our analysis we distinguish the four causes of death groups that comprise the largest number of deaths in Sweden. Data are coded following the 10th revision of the International Statistical Classification of Diseases (ICD). Table 1 provides the details on the classification of the four different causes of death groups that are distinguished here in the order of their corresponding chapters in ICD-10 (*neoplasms*, *diseases of the circulatory system*, *respiratory diseases*, and *mental and behavioral disorders and diseases of the nervous system*). All causes that are not included in one of the four groups are part of the group *Not elsewhere*. The calculations were done for each single year between 1970 and 2008.

**Table 1:** Causes of death used and their specific ICD codes.

Cause of Death	ICD-8	ICD-9	ICD-10
Neoplasms	140–239	140–239	C00–D48
Mental and behavioural disorders and diseases of the nervous system	290–358	290–359	F01–G99
Diseases of the circulatory system	390–459	390–459	I00–I99
Respiratory diseases	460–519	460–519	J00–J98
Not elsewhere	000–139	001–139	A00–B99
	240–289	240–289	D50–E90
	360–389	360–389	H00–H95
	520–799	520–799	K00–T98
	800–999	800–999	V01–Y89

In the last part of the analysis we use the same extended decomposition method of Arriaga [16] but instead of using estimates for the age-specific contribution of each cause of death to total mortality as input data for the decomposition, we use for the first time estimates for the age-specific contribution of smoking related mortality to total mortality at each age. This estimate is also called the fraction of deaths that can be attributed to smoking, or simply attributable fraction. One method to obtain the attributable fractions was proposed by Peto et al. [18] and uses lung cancer death rates as an indicator of the population's past smoking exposure in combination with estimates for the cause-specific mortality difference between smokers and non-smokers from the Cancer Prevention Study II, a large prospective cohort study, mortality attributable to smoking is then calculated indirectly. Here we use some updated estimates of the smoking attributable fraction from Peto et al. [19]. Those estimates are provided for two crude age groups, ages 35–69 and ages 70+ and are considered to be 0 at ages below 35. We use a spline interpolation of the cumulative form of the distribution to obtain smooth estimates for 5-year age groups that match the original grouping. Spline functions are also used to extrapolate values beyond the year 2000. As this approach is rather crude we tested alternative specifications for the distribution of the attributable fraction by age, but no alternative changed the overall contribution of smoking to the differences in life expectancy substantially. The annually obtained fractions of death that can be attributed to smoking by 5-year age group are then used in the decomposition for each year of our observation period. The method provides an estimate for the number of years that smoking related mortality contributes to differences in life expectancy between Sweden and selected other countries.

## Results

**Figure 2:**
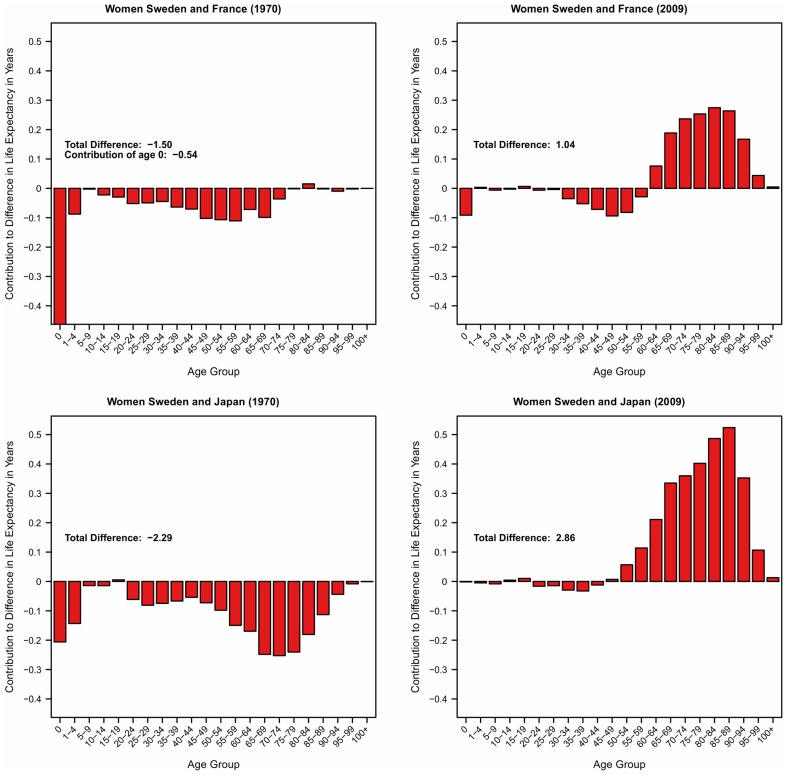
Age Contributions to Differences in Female Life Expectancy at Birth by Age Group (1970, 2009).

**Figure 3:**
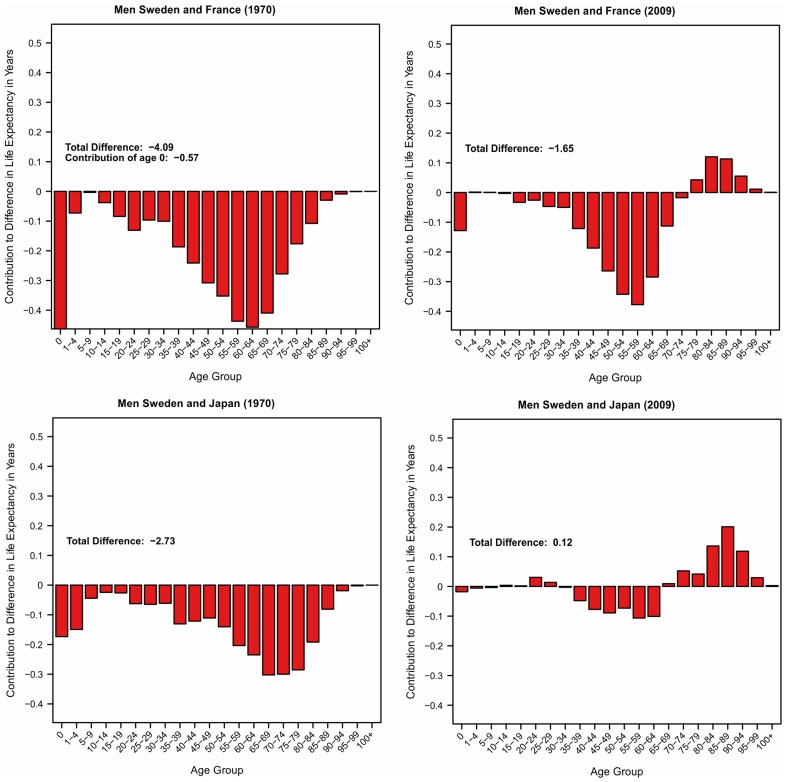
Age Contributions to Differences in Male Life Expectancy at Birth by Age Group (1970, 2009).

In the first part of our investigations we compared how age groups contribute to differences in life expectancy between Sweden and two of the leading countries in terms of life expectancy – Japan and France. The results for women are shown in Figure 2 and for men in Figure 3. Each panel provides the results for the years 1970 and 2009. Figures showing the results for all other years are available online (Animated Figure Set S1). Each individual bar gives the age-specific contribution to this overall life expectancy difference in years. Bars that are below 0 indicate a lower mortality for that age-category in Sweden compared to the other country and bars above 0 a higher mortality for Sweden. The sum of all individual bars is the total difference in life expectancy at birth between Sweden and the country of comparison.

Figure 2 shows that female Swedish life expectancy was the highest in the 1970s. In 1980 it was the same as in Japan and slightler higher than in France (0.4 years). For infant and child mortality Sweden has even been the leader. The contributions of the other ages to the differences are varying. At adult ages (15–64) Swedish mortality has been lower than in France and slightly higher than in Japan in 1980. At older ages (65+) Swedish women experienced lower mortality than Japanese women but higher mortality levels than French women. Sweden continued to have the lowest infant and child mortality from the 1980s until 2009 and mortality rates similar or slightly lower than in the other countries at ages below 50. However, above age 50 and especially at ages above 65 the pattern has changed and Swedish women experience considerably higher mortality than their French, and Japanese counterparts today.

Figure 3 provides the results for men. Similar to women, Swedish men experienced much higher life expectancy than Japan and France in the 1970s. In 1980, Swedish men had still much higher life expectancy than their French counterparts but lacked behind Japanese men by 0.8 years. Swedish infant and child mortality has also been lower than in France and Japan when considering only men. In 2009, Swedish men still experience lower mortality among infants and children, although the advance became smaller with time. At adult ages (15–64) Sweden is still a leading country, having considerably lower mortality than French and Japanese men. At old and very old ages, Swedish men are considerably disadvantaged, having substantially higher mortality than their French and Japanese counterparts. This mortality crossover is particularly remarkable when compared to French men. Swedish male life expectancy at birth is 1.6 years higher than male life expectancy in France. Despite this considerable overall advantage Swedish men are only doing better at ages below 65. The contribution of those ages amount to about 2 years of higher life expectancy in Sweden. However, French men do considerably better at ages above 65 so the overall advantage in life expectancy is reduced to 1.6 years.

In summary, for ages up to 65 Swedish men and women still experience age-specific mortality rates that are lower than the rates observed in France and Japan. At older ages the situation is different. Starting at about age 65 the decrease in mortality has been faster in Japan and France. Swedish mortality is considerably lagging behind, at increasing distance and especially for women.

**Figure 4:**
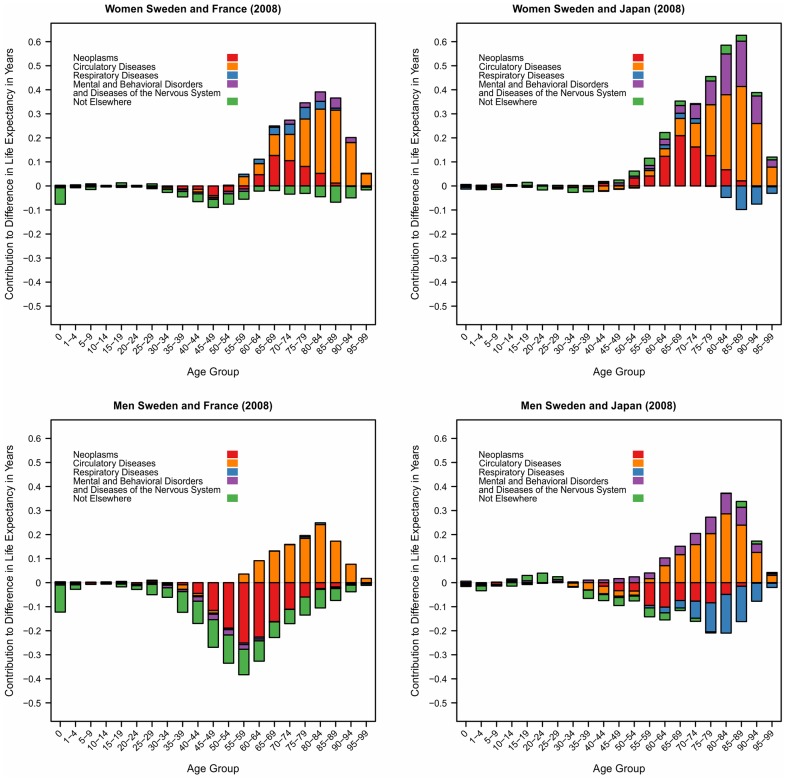
Cause of Death Contributions to Differences in Life Expectancy at Birth by Age Group (2008).

In the second step of the analysis we calculated the contributions of broad causes of mortality to life expectancy differences for each single year between 1970 and 2008. The results for the most recent year are presented in Figure 4, figures showing the results for all other years are available online (Animated Figure Set S2). Table 2 summarizes the contribution of broad causes of death for selected years. For women, the decomposition into broad causes of death shows that already in 1970 mortality from cancer was consistently higher in Sweden than in the other countries, while the mortality from cardiovascular diseases showed a less consistent pattern. For all other causes mortality was lower Sweden. Deaths from causes that were categorized *not elsewhere* were the main reason why Swedish female life expectancy was higher in the 1970s and 80s. Since then, the survival advantage from causes that were categorized *not elsewhere* has largely decreased. In 2008, mortality of Swedish women above age 60 was consistently higher for almost all causes of death (see Figure 4). Mortality from respiratory disease is the only exception when comparing Swedish women with their Japanese counterparts. Compared to French women, only mortality causes there were categorized *not elsewhere* has remained lower in Sweden.

**Table 2:** Cause of Death Contributions to Differences in Life Expectancy in 1970, 1990, 2008.

		Contribution in Years to Difference in Life Expectancy between…			
Year	Cause of Death	… Women in Sweden and France	… Women in Sweden and Japan	… Men in Sweden and France	… Men in Sweden and Japan
1970	Neoplasms	0.12	0.26	−1.06	−0.36
	Circulatory Diseases	0.72	−0.67	0.54	−0.38
	Respiratory Diseases	−0.08	−0.44	−0.32	−0.53
	Mental/Behavioral/Nervous System ^1)^	−0.24	−0.04	−0.28	0.04
	Not elsewhere	−2.01	−1.40	−2.97	−1.51
	All Causes ^2)^	−1.50	−2.29	−4.09	−2.73
1990	Neoplasms	0.11	0.51	−1.68	−0.54
	Circulatory Diseases	1.21	0.79	1.53	1.70
	Respiratory Diseases	0.07	−0.22	−0.07	−0.45
	Mental/Behavioral/Nervous System ^1)^	−0.03	0.28	−0.08	0.33
	Not elsewhere	−1.03	−0.03	−1.90	0.11
	All Causes ^2)^	0.34	1.34	−2.20	1.15
2008	Neoplasms	0.31	0.80	−1.26	−0.60
	Circulatory Diseases	1.24	1.41	1.11	1.12
	Respiratory Diseases	0.19	−0.21	−0.02	−0.69
	Mental/Behavioral/Nervous System ^1)^	0.10	0.76	−0.12	0.49
	Not elsewhere	−0.52	0.17	−1.24	−0.12
	All Causes ^2)^	1.32	2.93	−1.53	0.21

1) Abbreviation of “Mental and Behavioral Disorders and Diseases of the Nervous System”

2) The contribution of All Causes in years is the total difference in life expectancy and calculated as the sum of the cause-specific contributions.

For Swedish men the contributions of broad causes of deaths have been quite stable over time. In 1970, both Japanese and French man had a substantially lower life expectancy than Swedish men. Compared to Japanese men, all causes of death contributed to this overall advantage except for deaths from *Mental and Behavioral Disorders and Diseases of the Nervous System*, which had no impact. Swedish men also had consistently lower mortality than French men from all broad causes of death except *Circulatory Diseases*. Figure 4 shows that the contribution of causes of death between the life expectancy of Swedish and French men remained very stable over time. As in 1970, all causes except for mortality from cardiovascular diseases contribute to the survival advantage of Swedish men. The negative impact of cardiovascular mortality is high and sums up to more than 1 year with large negative contributions between the age 60 and 94. An almost similar contribution can be found when comparing Swedish men to their Japanese counterparts. At the same time cancer mortality is consistently lower in Sweden with large positive contributions between the ages 40 and 85. Recently, deaths from mental and behavioural disorders (mainly dementia) especially at old age began to contribute somewhat negatively, indicating that Swedish mortality from these diseases is lagging behind the other countries.

**Figure 5:**
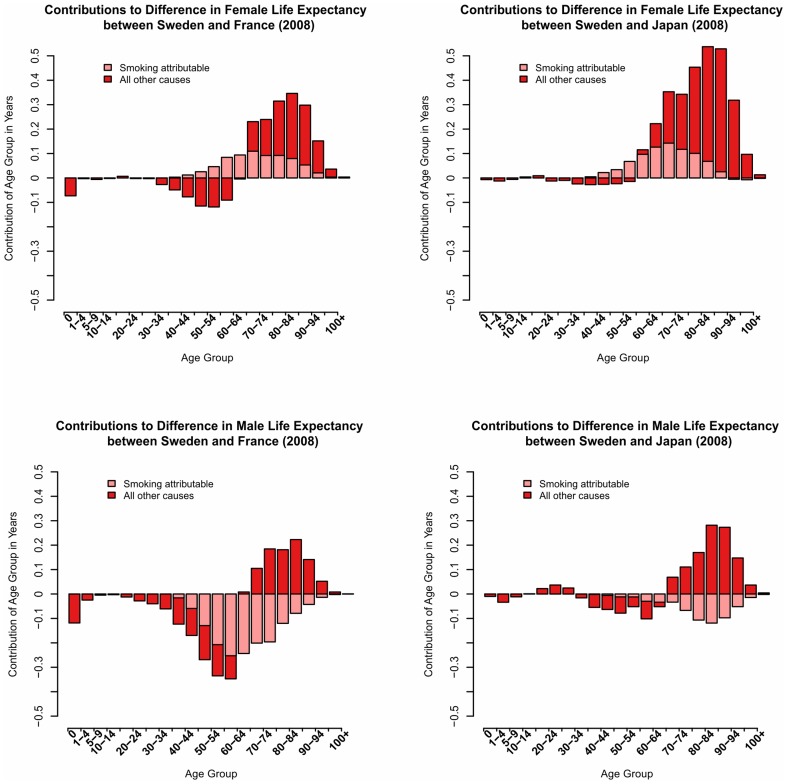
Contributions of Smoking Attributable Mortality to Differences in Life Expectancy at Birth by Age (2008).

In the last step of the analysis we assess the impact of different smoking habits on the observed mortality pattern for men and women. Figure 5 shows a similar set of figures as before but gives the estimated contribution of smoking to the life expectancy differences in 2008. Figures that present the results for the years 1970 to 2008 are available online (Animated Figure Set S3). Table 3 summarizes the contribution of death attributed to smoking for selected years. In 1970 smoking contributed very little to the life expectancy differences between Swedish, Japanese, and French women, whilethe contribution of smoking to life expectancy differences became a little more substantial between Swedish and French women in 1990. Overall the contribution tended to be higher in Sweden than in the other countries. With time the impact of smoking increased and contributed a higher share to life expectancy differences. In 2008, smoking related mortality for women was higher in Sweden than in the other two countries. It is estimated that about half of the lower life expectancy of Swedish women as compared to their French counterparts is due to smoking (0.72 years). The contribution of smoking in years is quite similar when compared with Japanese women, however, its relative contribution to the overall difference has remained relatively modest (0.79 years, corresponding to 27% of the overall difference in life expectancy). The contribution is even lower at the oldest ages where Swedish excess mortality is particularly pronounced.

**Table 3:** Contributions of Smoking Attributable Mortality to Differences in Life Expectancy in 1970, 1990, 2008.

		Contribution in Years to Difference in Life Expectancy between…			
Year	Cause of Death	… Women in Sweden and France	… Women in Sweden and Japan	… Men in Sweden and France	… Men in Sweden and Japan
1970	Attributable to smoking	0.09	−0.10	−1.54	−0.02
	Not attributable to smoking	−1.58	−2.19	−2.55	−2.71
	All Causes ^1)^	−1.50	−2.29	−4.09	−2.73
1990	Attributable to smoking	0.47	0.20	−1.94	−0.10
	Not attributable to smoking	−0.13	1.14	−0.26	1.25
	All Causes ^1)^	0.34	1.34	−2.20	1.15
2008	Attributable to smoking	0.72	0.79	−1.56	−0.59
	Not attributable to smoking	0.61	2.15	0.03	0.80
	All Causes ^1)^	1.32	2.94	−1.53	0.21

Notes: Negative values indicate higher life expectancy in Sweden than in the country of comparison, and vice versa.

1) The contribution of All Causes in years is the total difference in life expectancy and calculated as the sum of the cause-specific contributions.

For men, smoking contributed very differently when comparing to Japan and when comparing to France. At the beginning of the 1970s deaths attributable to smoking seem to have been a major reason for the higher life expectancy of Swedish men as compared to their French counterparts, while its contribution was very small when compared with Japanese men. With time the negative impact increased and thus In 2008, mortality due to smoking is noticeably higher in Japanese men than in Swedish men and sums up to about 0.6 years difference in life expectancy. In spite of the positive impact of smoking overall male life expectancy still remains slightly lower in Sweden than in Japan. The smoking excess mortality of French men persisted over time and sums up to almost 1.5 years in lower life expectancy as compared to their Swedish counterparts. Despite the large positive contribution of smoking up to the highest ages, the overall contribution of the ages above 75 has been negative. In summary, we found that smoking attributable mortality is consistently lower in Swedish men and an important contributor to life expectancy. Its substantial positive impact is dampened by the higher mortality from causes unrelated to smoking, especially at higher ages.

## Discussion

In this study we have observed how Sweden loses ground compared to other countries with respect to life expectancy. Using decomposition techniques we examined this development in detail comparing the Swedish mortality to France and Japan, two of the leading countries in terms of life expectancy for each single year between 1970 and 2008. From the beginning of the 20^th^ century until the 1970s Sweden had a higher life expectancy than both countries and all ages contributed to this advantage. Since then Sweden has lost its relative advantage, especially for women, because mortality at older ages improved more slowly than in the now leading countries. Today, it is only among infants, children and young adults that Sweden is the leading country. At ages above 65 years Swedish mortality is lagging behind at an increasing distance, although it continues to improve. To shed some light on the underlying mechanisms we explored both the impact of different causes of death as well as the contribution of deaths attributable to smoking. Our analyses showed that the unfavorable trends in Sweden are largely due to cardiovascular disease mortality. Trends in cancer mortality also play a role for women, whereas cancer mortality of Swedish men is substantially lower than that of French and Japanese men. In recent years, the contribution of mental and behavioral disorders and diseases of the nervous system has increased and tends to be higher in Sweden than in the other countries for both men and women. The differences are especially pronounced at the highest ages and likely reflect mortality differences from degenerative diseases such as dementia. The contribution of other causes of deaths has been inconsistent.

This study is the first to use a decomposition method to analyze how smoking related deaths contribute to life expectancy differences between countries. In men smoking rates and smoking related mortality have always been very low and therefore are a substantial contributor to a more positive development. However, despite the fact that the particular low smoking levels in Swedish men should contribute to a lower mortality from cardiovascular diseases, mortality from these causes is still much higher compared to men in France and Japan. This suggests that factors not related to smoking contribute to the Swedish excess mortality from cardiovascular diseases. Swedish women lag behind the men with respect to smoking and trends in smoking rates may provide a partial explanation for the trends in women, however, it is not possible to isolate one single explanatory factor for why Sweden is losing ground. One important contribution may be the lower longevity of less educated women in Sweden than in France [20]. A potential limitation in the analyses of the contribution of different causes of death is the lower quality of cause of death data at the highest ages. At these ages comorbidity is very common and misclassifications are more likely to occur than at younger ages. This may be a problem in all of the studied countries; however, the extent may vary due to differences in autopsy rates and the diagnostic quality in general. In the case that the accuracy of cause of death information is better in one country than in another, our estimates for the relative contribution of a cause to overall life expectancy could be systematically under- or overestimated. Our data also included cause of death from different revisions of the ICD; however, for large groups, as used here, translation from one revision to another is normally not problematic. A further limitation is the use of indirect estimates for the contribution of deaths attributable to smoking using the Peto-Lopez method. According to this method, deaths from external causes, neonatal deaths, deaths from liver cirrhosis, and all deaths under age 35 are never considered to be due to smoking. Furthermore, the method aims to obtain conservative estimates for the attributable fraction by decreasing the excess mortality of smokers by half for many diseases. As a consequence, the method tends to rather underestimate smoking attributable mortality for each specific country and eventually also the impact of smoking on life expectancy differences between two countries. However, other methods for obtaining the attributable fraction, such as the regression based approach by Preston et al. [21], have generally confirmed the validity of the estimates from the Peto-Lopez method; therefore the impact of the underestimations is probably rather small. In addition it is assumed that when comparing death rates among smokers and non-smokers other risk factors are equally distributed among the two groups. This might not be the case; however, as long as this is similar between the countries that we compare, it should not have a large impact on the relative contribution of smoking related deaths between Sweden and the other two countries. The decomposition method used here does not address whether cohort effects may shape some of the observed differences. To address this issue we conducted additional decompositions comparing cohort mortality schedules; the results did not differ substantially and did not provide additional insights.

We conclude that progress in the reduction of old-age mortality has slowed in Sweden compared to other leading countries. At ages above 100 we even observe a complete lack of mortality reduction in Sweden, while other countries continue to improve [3], [4]. Although changes in the trends for cardiovascular diseases are the biggest contributors to this, it is not possible to point out one single explanation. The reductions that have been seen in cardiovascular mortality in the past have partly been due to a reduced incidence of the disease and partly due to improved survival for those with the disease. It is not clear at present which of these resulted in a lower rate of progress. We now know how Sweden compares to two leading countries, and future studies need to establish whether the observed pattern in Sweden is unique or whether modest reductions in old-age mortality and very few reductions at very-old age is becoming a more widespread pattern. Future research also needs to take into account factors that might shape the observed divergences such as differences in the care of the elderly.

## Supporting Information

Appendix S1 **Decomposition formulas by Arriaga.**
(DOCX)

Animated Figure Set S1 **Age Contributions to Differences in Life Expectancy at Birth by Age Group and Sex (1970–2009).**
(PDF)

Animated Figure Set S2 **Cause of Death Contributions to Differences in Life Expectancy at Birth by Age Group and Sex (1970–2008).**
(PDF)

Animated Figure Set S3 **Contributions of Smoking Attributable Mortality to Differences in Life Expectancy at Birth by Age and Sex (1970–2008).**
(PDF)
