# Supplementary material for: Losing Ground - Swedish Life Expectancy in a Comparative Perspective
Source: PLoS One. 2014 Feb 6;9(2):e88357. doi: 10.1371/journal.pone.0088357 (PMC3916411; doi:10.1371/journal.pone.0088357)
Supplement: Appendix S1 — Decomposition formulas by Arriaga. (DOCX) [file pone.0088357.s001.docx]

**Appendix S1A**

The decomposition formula by Arriaga [14] is

$${{}_{n}\triangle}_{x}=\frac{l_{x}^{1}}{l_{o}^{1}}\cdot\left( \frac{{{}_{n}L}_{x}^{2}}{l_{x}^{2}}-\frac{{{}_{n}L}_{x}^{1}}{l_{x}^{1}} \right)+\frac{T_{x+n}^{2}}{l_{0}^{1}}\cdot\left( \frac{l_{x}^{1}}{l_{x}^{2}}-\frac{l_{x+n}^{1}}{l_{x+n}^{2}} \right)$$

where ${{}_{\boldsymbol{n}}\boldsymbol{\triangle}}_{\boldsymbol{x}}$ gives the contribution of the all-cause mortality difference in age group x to x+n to differences in life expectancy at birth between two populations in years. The method is based on conventional life table functions $\boldsymbol{l}_{\boldsymbol{x}}$, $\boldsymbol{T}_{\boldsymbol{x}}$ , and ${{}_{\boldsymbol{n}}\boldsymbol{L}}_{\boldsymbol{x}}$.

**Appendix S1B**

The formula for the extension is

$${}_{n}{\triangle_{x}^{i}}={{}_{n}\triangle}_{x}\cdot\frac{{{}_{n}R}_{x}^{i}\left( 2 \right)\cdot{{}_{n}m}_{x}\left( 2 \right)-{{}_{n}R}_{x}^{i}\left( 1 \right)\cdot{{}_{n}m}_{x}\left( 1 \right)}{{{}_{n}m}_{x}\left( 2 \right)-{{}_{n}m}_{x}\left( 1 \right)}$$

Where ${}_{n}{\triangle_{x}^{i}}$ gives the number of years that a cause of death $i$ is contributing to differences in life expectancy between two populations. Here ${}_{n}{m_{x}}$ is the mortality rate between ages $x$ and $x+n$ and ${{}_{n}R}_{x}^{i}\left( j \right)$ is the proportion of deaths from cause $i$ in age group x to x+n in population $j$. As shown in equation 1, ${{}_{n}\triangle}_{x}$ is the all-cause mortality difference in age group x to x+n.
