# Supplementary material for: Losing Ground - Swedish Life Expectancy in a Comparative Perspective
Source: PLoS One. 2014 Feb 6;9(2):e88357. doi: 10.1371/journal.pone.0088357 (PMC3916411; doi:10.1371/journal.pone.0088357)
Supplement: Animated Figure Set S1 — Age Contributions to Differences in Life Expectancy at Birth by Age Group and Sex (1970–2009). (PDF) [file pone.0088357.s002.pdf]

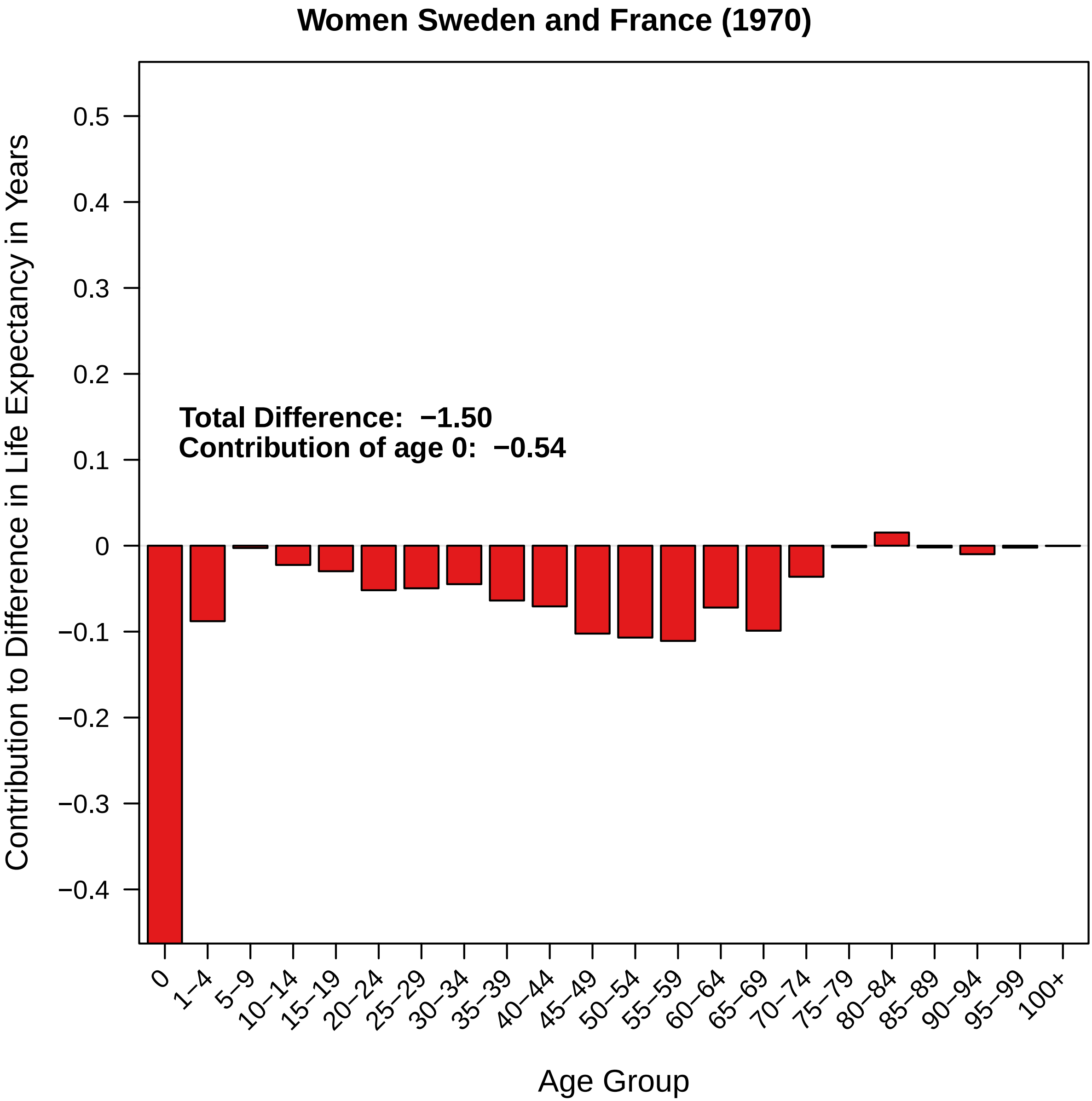

Women Sweden and France (1970)
Total Difference: −1.50
Contribution of age 0: −0.54
Contribution to Difference in Life Expectancy in Years
0.5
0.4
0.3
0.2
0.1
0
−0.1
−0.2
−0.3
−0.4
0
1−4
5−9
10−14
15−19
20−24
25−29
30−34
35−39
40−44
45−49
50−54
55−59
60−64
65−69
70−74
75−79
80−84
85−89
90−94
95−99
100+
Age Group

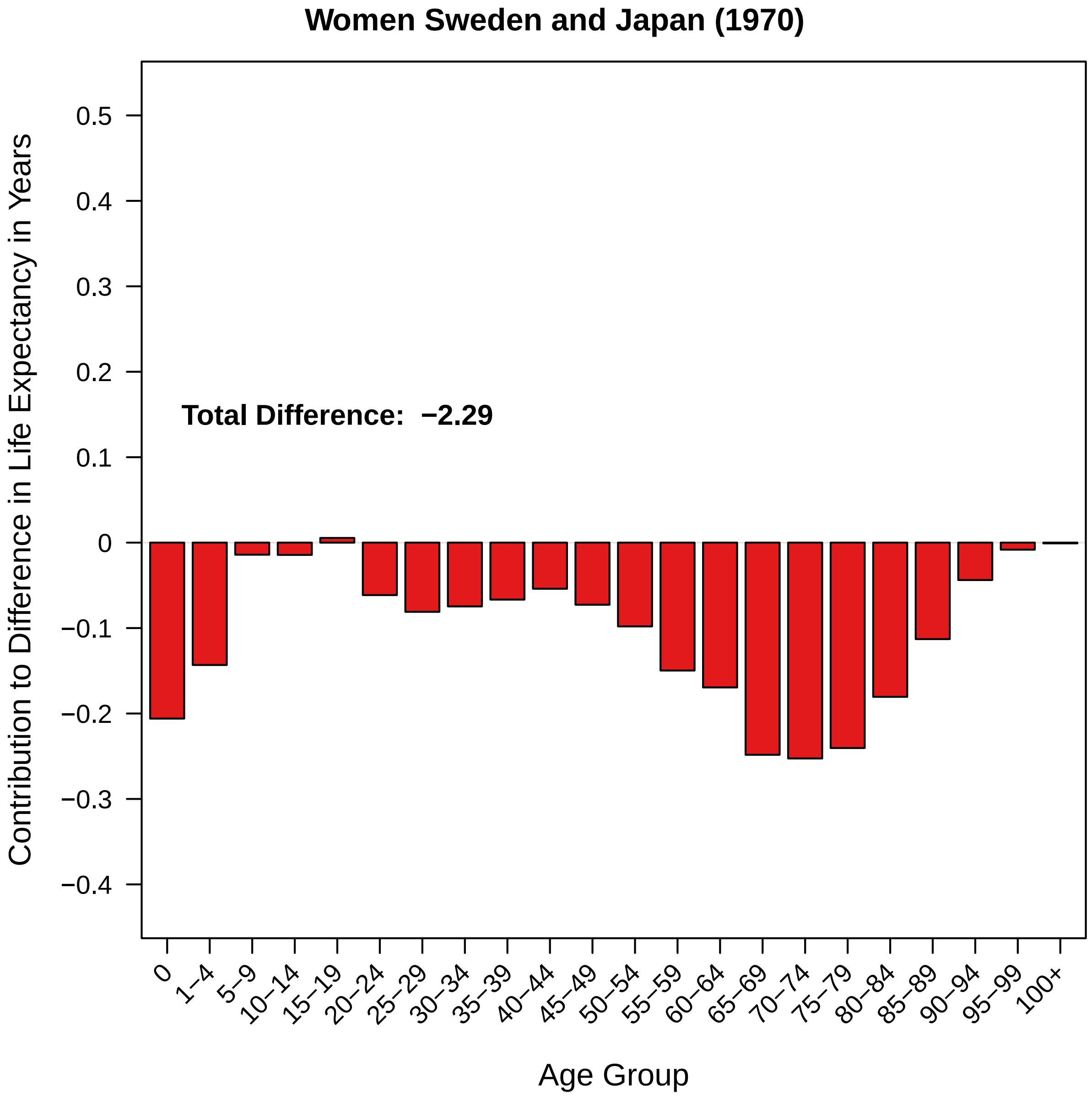
Women Sweden and Japan (1970)
Contribution to Difference in Life Expectancy in Years
0.5
0.4
0.3
0.2
0.1
0
−0.1
−0.2
−0.3
−0.4
Total Difference: −2.29
0
1−4
5−9
10−14
15−19
20−24
25−29
30−34
35−39
40−44
45−49
50−54
55−59
60−64
65−69
70−74
75−79
80−84
85−89
90−94
95−99
100+
Age Group

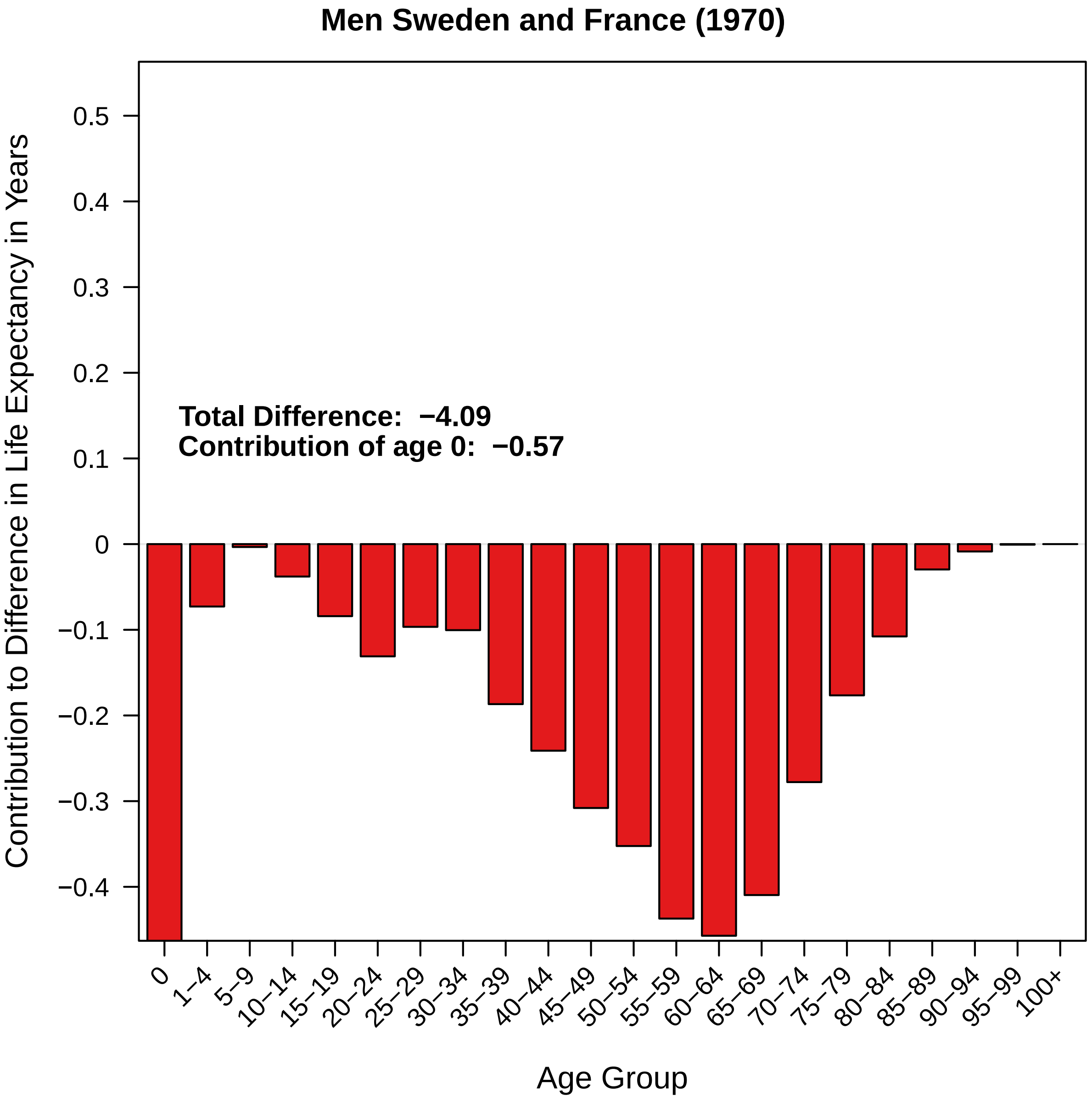
Men Sweden and France (1970)
Total Difference: −4.09
Contribution of age 0: −0.57
Contribution to Difference in Life Expectancy in Years
0.5
0.4
0.3
0.2
0.1
0
−0.1
−0.2
−0.3
−0.4
0
1−4
5−9
10−14
15−19
20−24
25−29
30−34
35−39
40−44
45−49
50−54
55−59
60−64
65−69
70−74
75−79
80−84
85−89
90−94
95−99
100+
Age Group

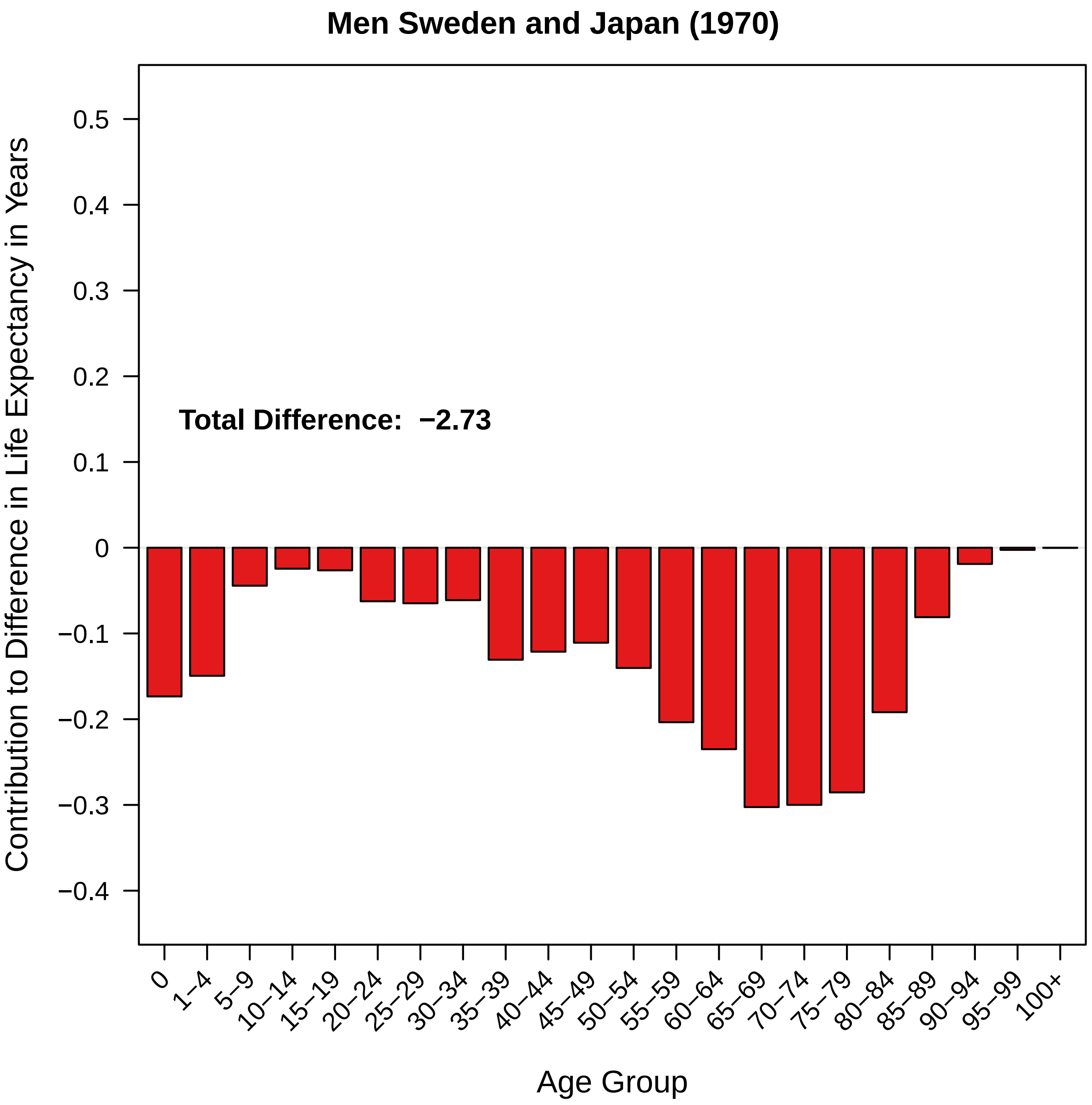

Men Sweden and Japan (1970)
Total Difference: −2.73
Contribution to Difference in Life Expectancy in Years
0.5
0.4
0.3
0.2
0.1
0
−0.1
−0.2
−0.3
−0.4
0
1−4
5−9
10−14
15−19
20−24
25−29
30−34
35−39
40−44
45−49
50−54
55−59
60−64
65−69
70−74
75−79
80−84
85−89
90−94
95−99
100+
Age Group
