# Supplementary material for: Losing Ground - Swedish Life Expectancy in a Comparative Perspective
Source: PLoS One. 2014 Feb 6;9(2):e88357. doi: 10.1371/journal.pone.0088357 (PMC3916411; doi:10.1371/journal.pone.0088357)
Supplement: Animated Figure Set S2 — Cause of Death Contributions to Differences in Life Expectancy at Birth by Age Group and Sex (1970–2008). (PDF) [file pone.0088357.s003.pdf]

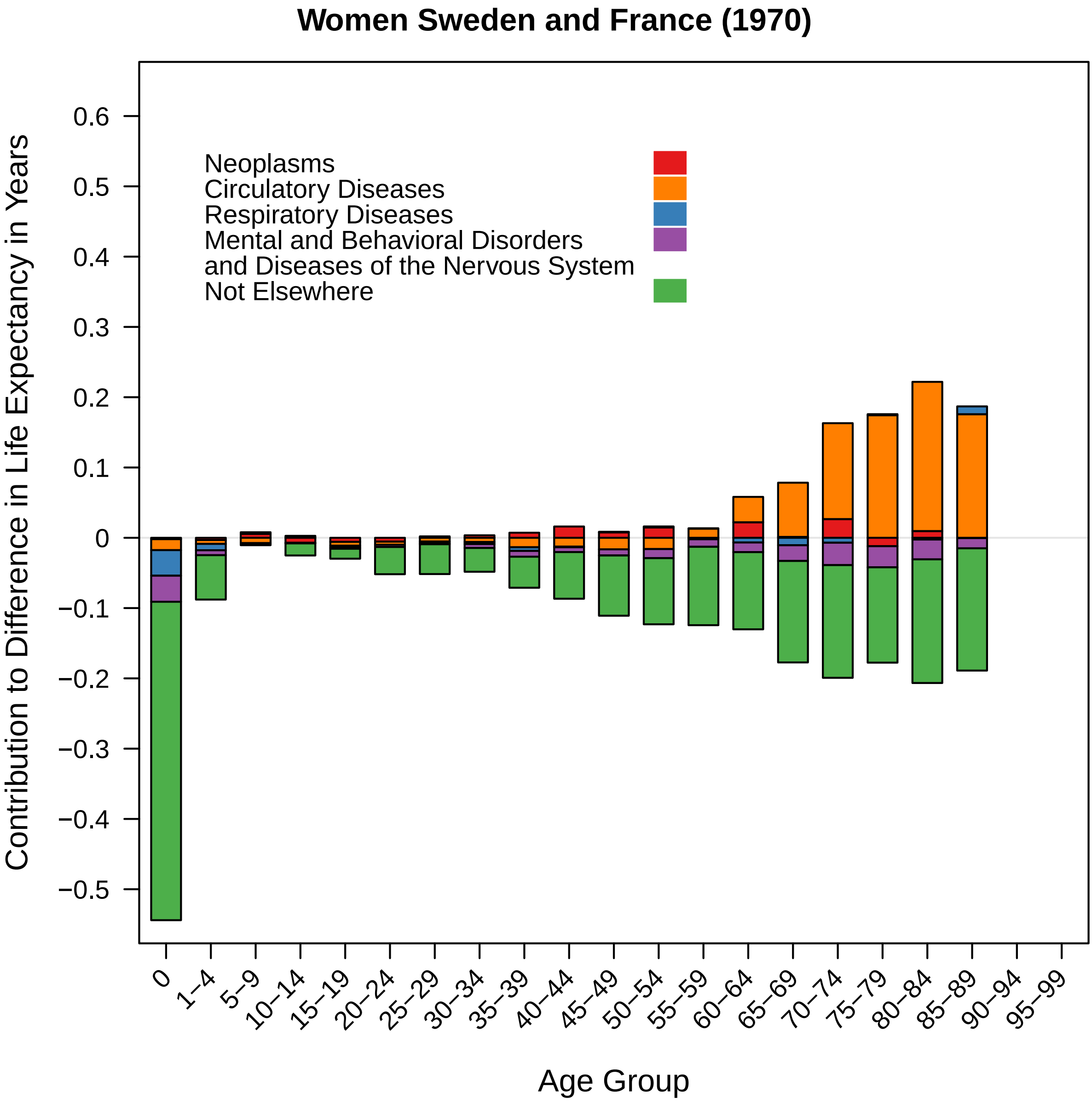
Women Sweden and France (1970)
Neoplasms
Circulatory Diseases
Respiratory Diseases
Mental and Behavioral Disorders
and Diseases of the Nervous System
Not Elsewhere
Contribution to Difference in Life Expectancy in Years
0.6
0.5
0.4
0.3
0.2
0.1
0
−0.1
−0.2
−0.3
−0.4
−0.5
0
1–4
5–9
10–14
15–19
20–24
25–29
30–34
35–39
40–44
45–49
50–54
55–59
60–64
65–69
70–74
75–79
80–84
85–89
90–94
95–99
Age Group

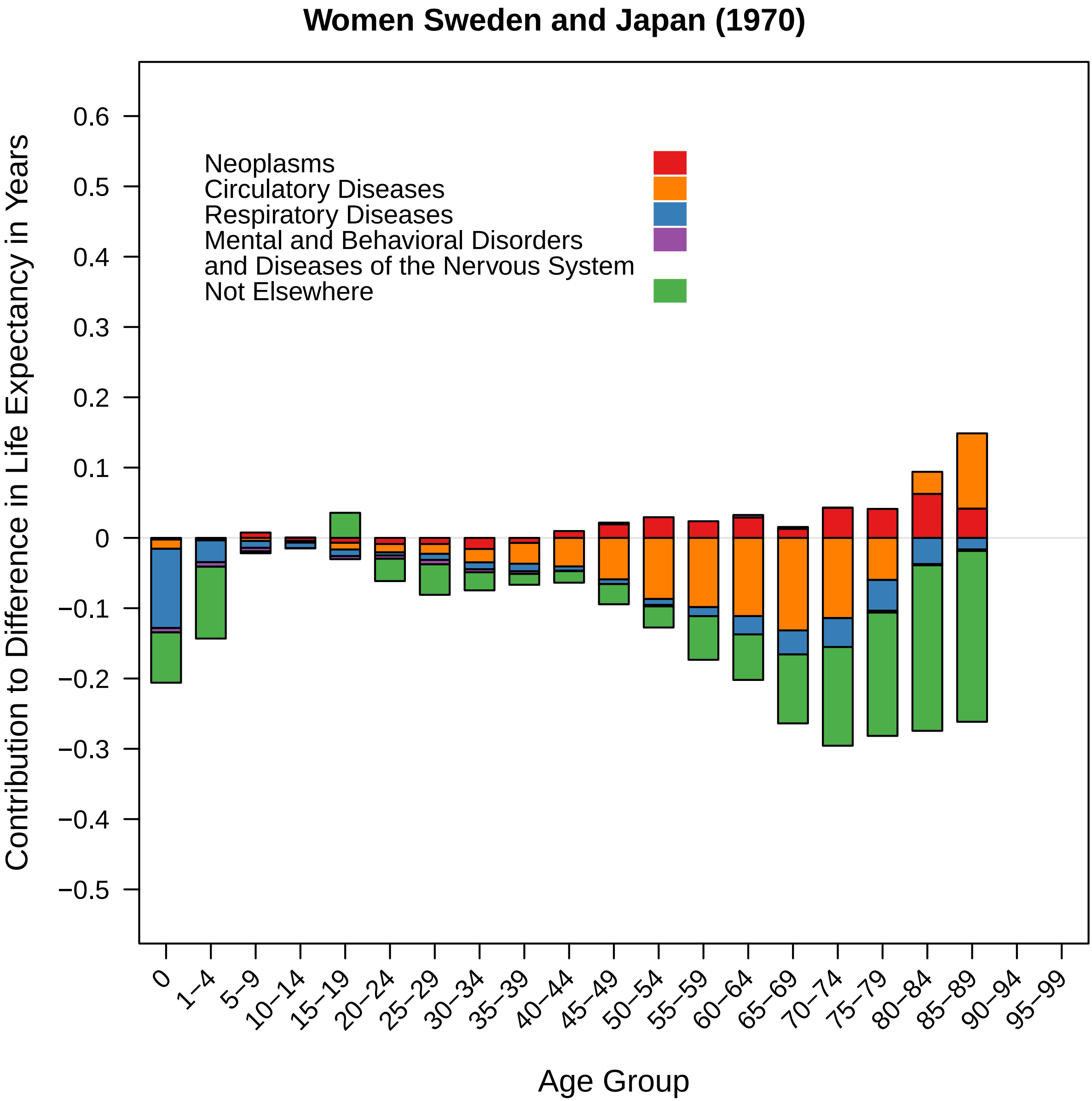
Women Sweden and Japan (1970)
Neoplasms
Circulatory Diseases
Respiratory Diseases
Mental and Behavioral Disorders
and Diseases of the Nervous System
Not Elsewhere
Contribution to Difference in Life Expectancy in Years
0.6
0.5
0.4
0.3
0.2
0.1
0
−0.1
−0.2
−0.3
−0.4
−0.5
0
1−4
5−9
10−14
15−19
20−24
25−29
30−34
35−39
40−44
45−49
50−54
55−59
60−64
65−69
70−74
75−79
80−84
85−89
90−94
95−99
Age Group

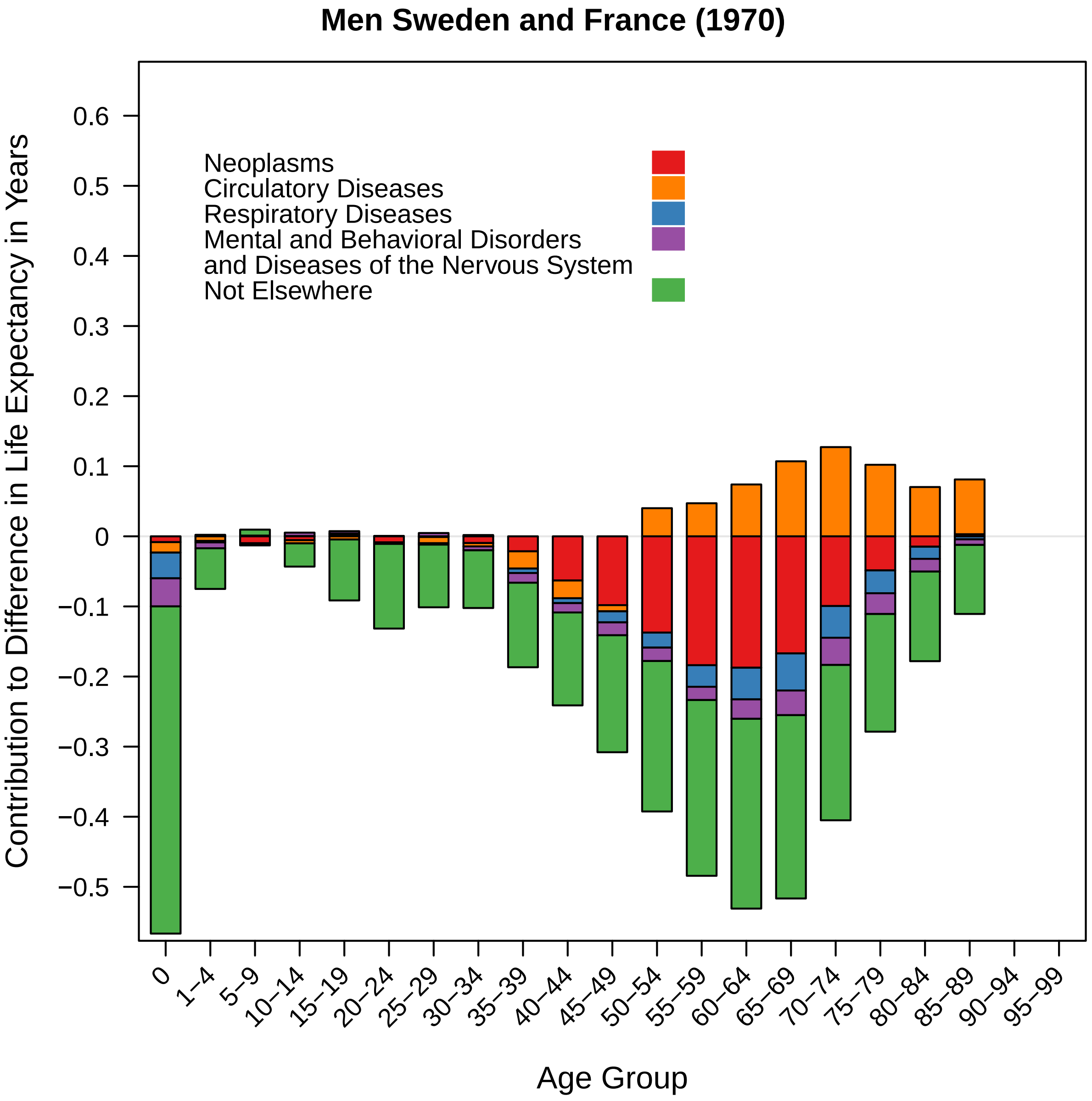
Men Sweden and France (1970)
Neoplasms
Circulatory Diseases
Respiratory Diseases
Mental and Behavioral Disorders
and Diseases of the Nervous System
Not Elsewhere
Contribution to Difference in Life Expectancy in Years
0.6
0.5
0.4
0.3
0.2
0.1
0
−0.1
−0.2
−0.3
−0.4
−0.5
0
1−4
5−9
10−14
15−19
20−24
25−29
30−34
35−39
40−44
45−49
50−54
55−59
60−64
65−69
70−74
75−79
80−84
85−89
90−94
95−99
Age Group

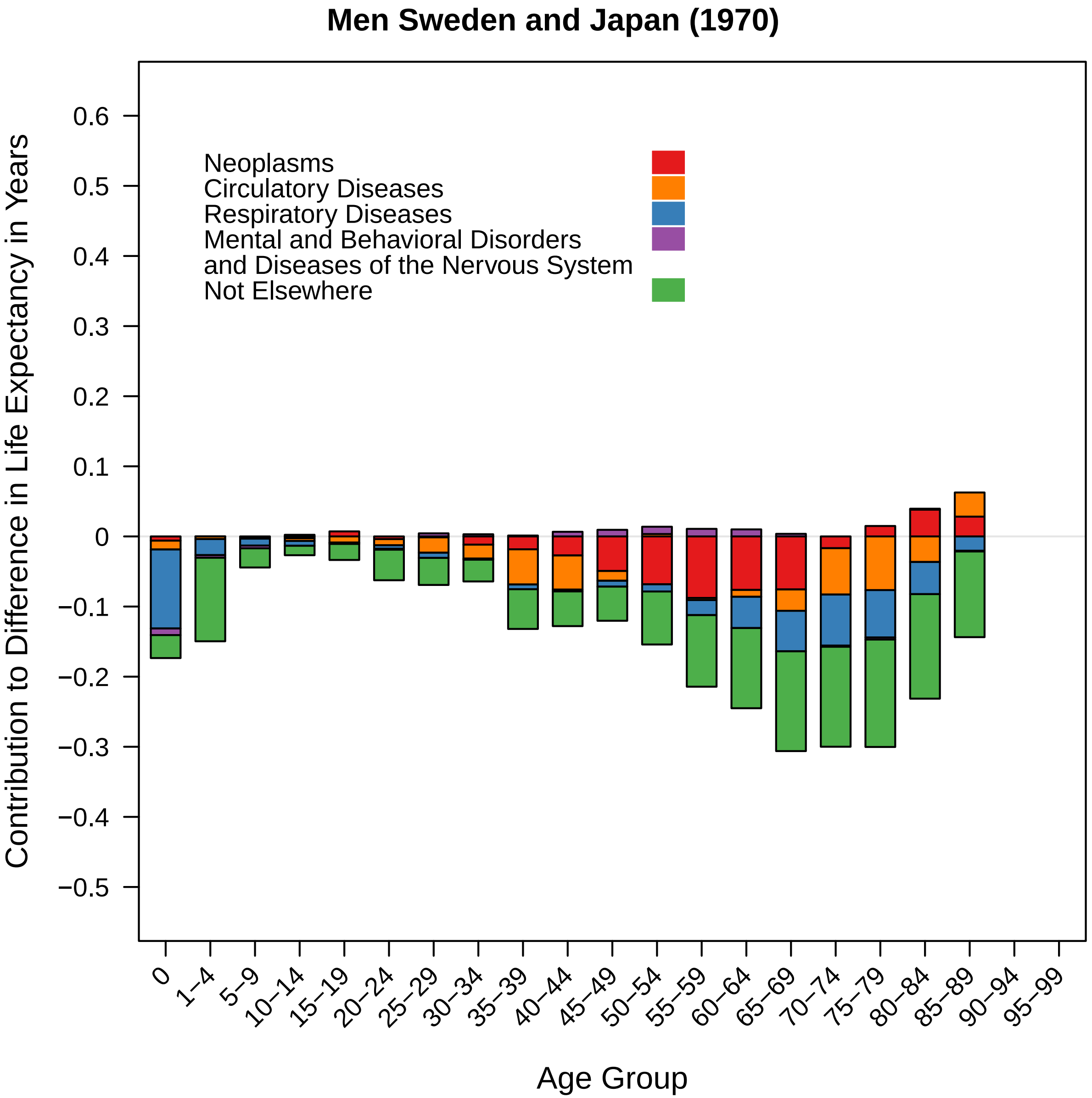

Men Sweden and Japan (1970)
Neoplasms
Circulatory Diseases
Respiratory Diseases
Mental and Behavioral Disorders
and Diseases of the Nervous System
Not Elsewhere
Contribution to Difference in Life Expectancy in Years
0.6
0.5
0.4
0.3
0.2
0.1
0
−0.1
−0.2
−0.3
−0.4
−0.5
0
1–4
5–9
10–14
15–19
20–24
25–29
30–34
35–39
40–44
45–49
50–54
55–59
60–64
65–69
70–74
75–79
80–84
85–89
90–94
95–99
Age Group
