# Supplementary material for: Losing Ground - Swedish Life Expectancy in a Comparative Perspective
Source: PLoS One. 2014 Feb 6;9(2):e88357. doi: 10.1371/journal.pone.0088357 (PMC3916411; doi:10.1371/journal.pone.0088357)

Contributions to Difference in Female Life Expectancy between Sweden and France (1970)

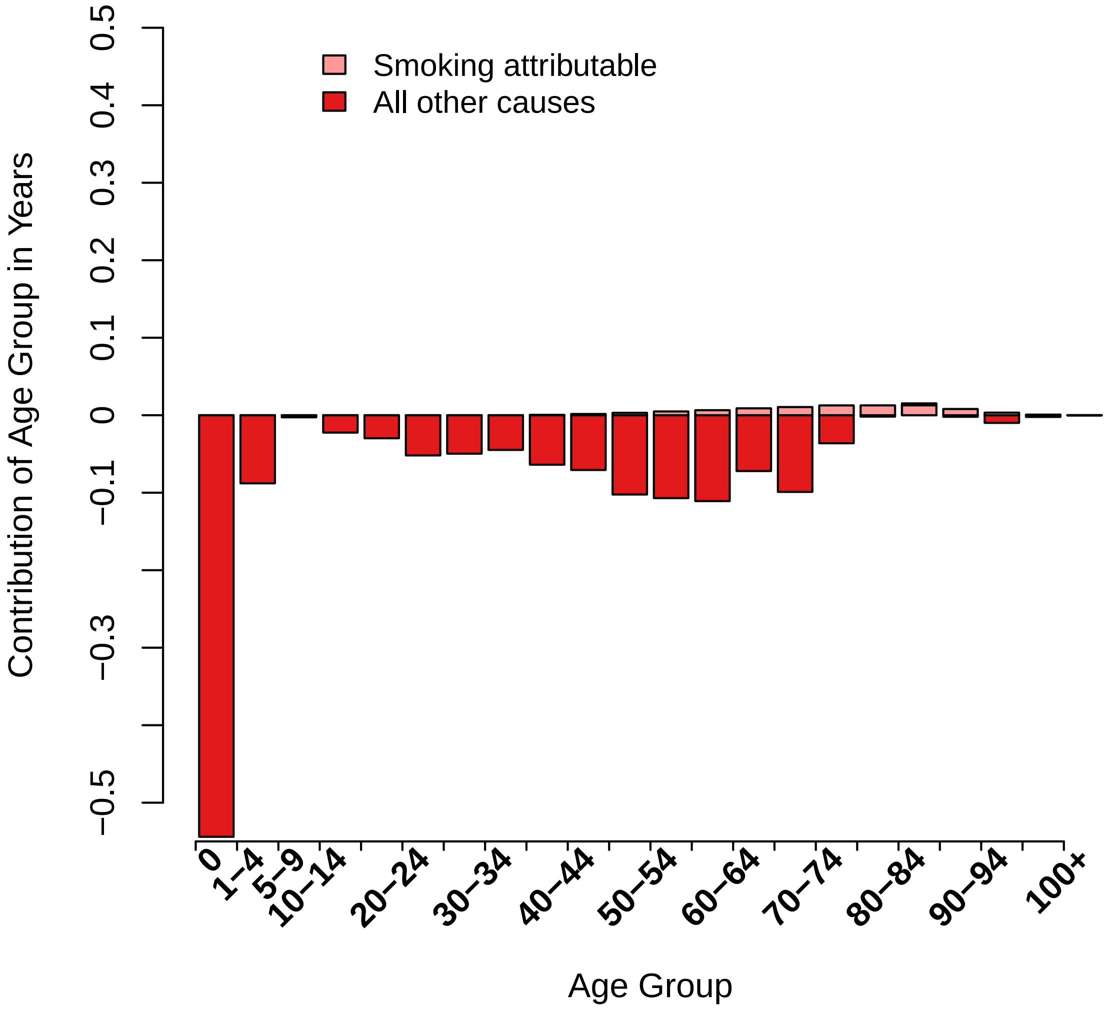

Contributions to Difference in Female Life Expectancy between Sweden and Japan (1970)

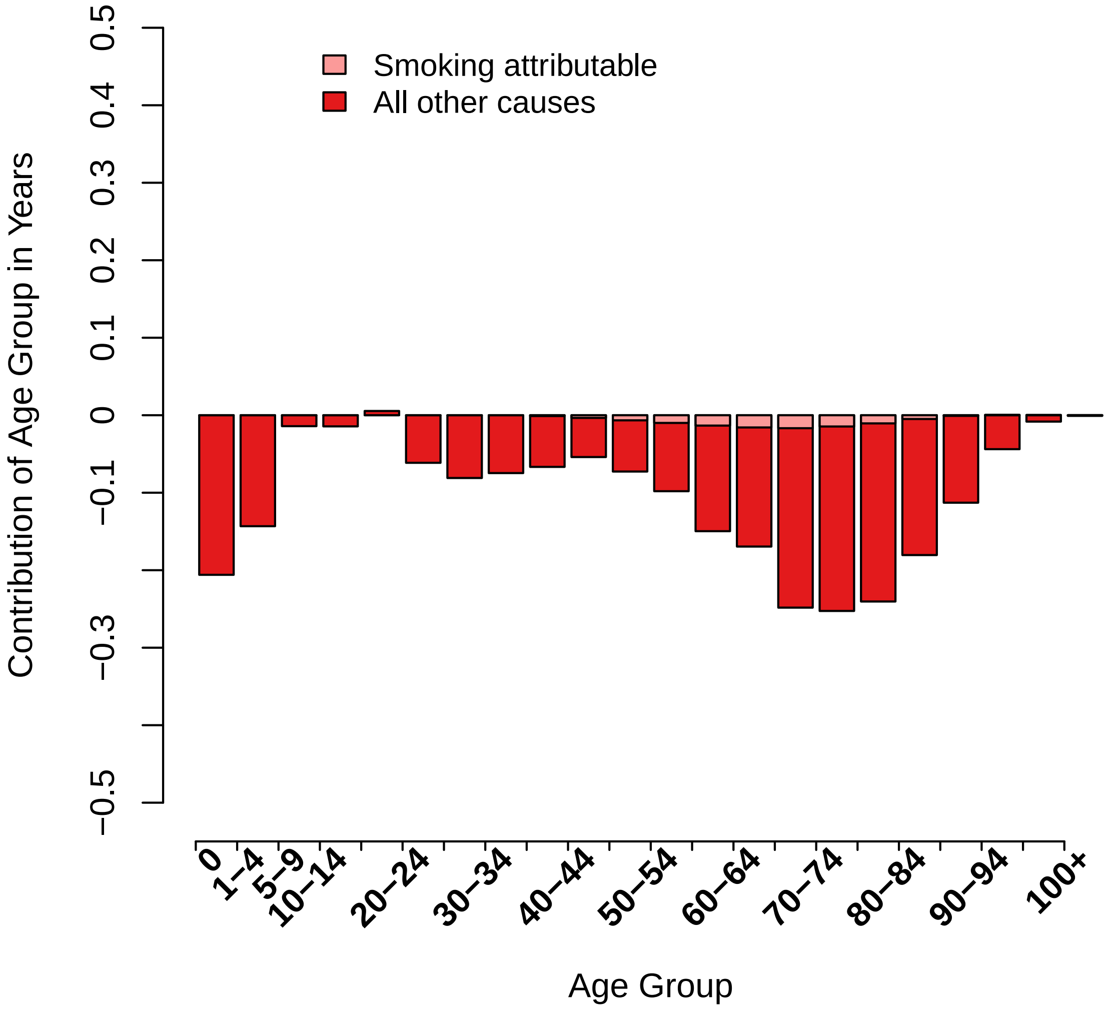

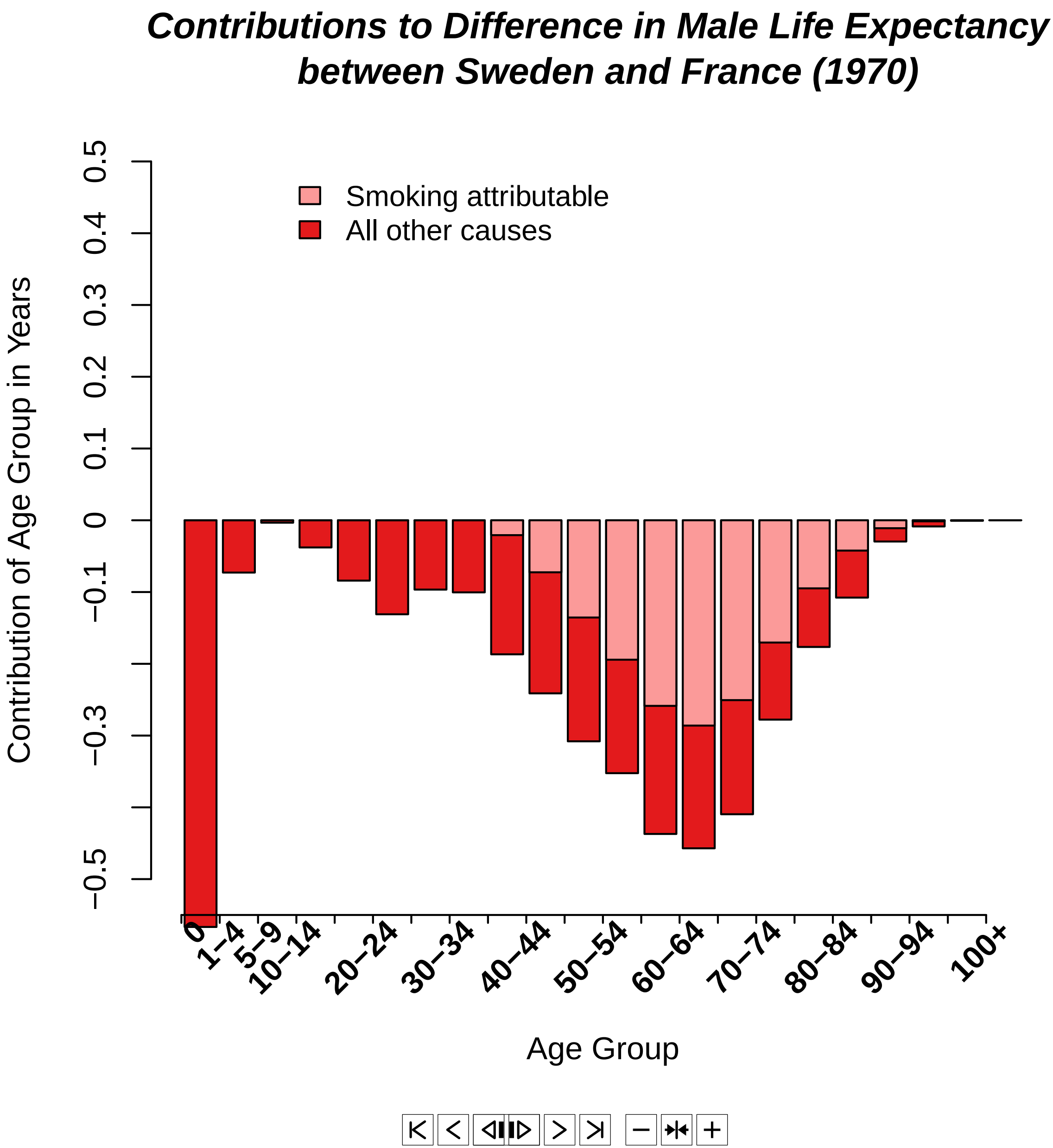

Contributions to Difference in Male Life Expectancy
between Sweden and France (1970)
Smoking attributable
All other causes
Contribution of Age Group in Years
0.5
0.4
0.3
0.2
0.1
0
−0.1
−0.3
−0.5
0
1−4
5−9
10−14
20−24
30−34
40−44
50−54
60−64
70−74
80−84
90−94
100+
Age Group

Contributions to Difference in Male Life Expectancy between Sweden and Japan (1970)

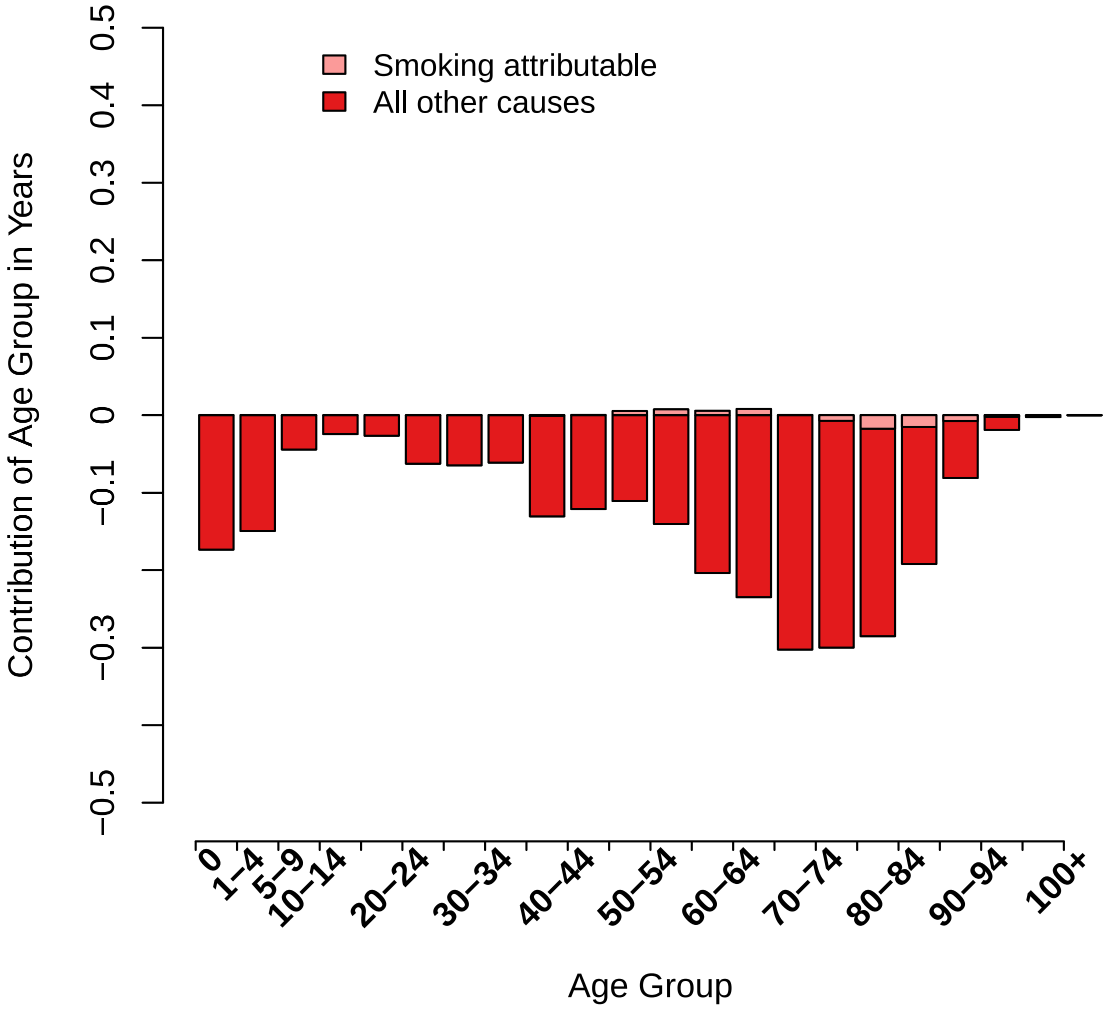

Supplement: Animated Figure Set S3 — Contributions of Smoking Attributable Mortality to Differences in Life Expectancy at Birth by Age and Sex (1970–2008). (PDF) [file pone.0088357.s004.pdf]
